# Antimicrobial use among under-five hospitalized children in Bangladesh: Findings from a Point Prevalence Survey

**DOI:** 10.1017/ash.2024.165

**Published:** 2024-09-16

**Authors:** Md. Golam Dostogir Harun, ABM Alauddin Chowdhury, Shariful Amin Sumon

**Affiliations:** International Centre for Diarrhoeal Disease Research, Bangladesh (Icddr,b); Dept. of Public Health, Daffodil Internation University

## Abstract

**Background:** The use of antibiotics and the occurrence of antimicrobial resistance in Bangladesh are very high. Inappropriate use of antibiotics among hospitalized children has contributed to a high rise in antimicrobial resistance in Bangladesh. Data on the rational use of antibiotics in Bangladeshi hospitals are limited. This study documented current antimicrobial usage among children under five in selected tertiary hospitals in Bangladesh. **Methods:** From August to September 2022, we conducted a point prevalence survey in four tertiary hospitals in Bangladesh. We used the World Health Organization’s Point Prevalence Survey (PPS) methods and guidelines to conduct the study. Study participants were hospitalized under the age of five years children, and we collected information from the pediatric and neonatal wards of each hospital. Antibiotic-suggesting shapes were analyzed according to WHO AWaRe metrics and Anatomical Therapeutic Chemical (ATC) Classification. **Results:** The assessment included 189 children under the age of five, with the majority (78.8%, 149/189) being under one year children. Approximately three-fourths (75.1%) of children had peripheral vascular catheters following admission. Overall, 86.2% (163/189) of children were given antibiotics after being admitted to the hospital, with infants receiving the most (81.0%, 132/163). The majority of antibiotics were administered by parents (84.7%). Antibiotics from the Watch Group were most commonly prescribed (73.0%, 119/163), followed by a combination of the Watch and Access Groups (23.3%) to treat the children. Ceftriaxone (63.8%), Meropenem (16.0%), and Ceftazidime/Amikacin (8.0%) were the most regularly prescribed antibiotics. Young children (< 1 year) were more likely to get antibiotics (AOR: 3.54, p-value: 0.003) than the other children under the age of five. **Conclusion:** The data showed that most children received empirical antibiotics during hospitalization, and overuse of broad-spectrum Watch group antibiotics was common practice in hospital settings. Developing and implementing antibiotic use guidelines is critical to limit the inappropriate use of antibiotics for young children

